# Reduced inattention and hyperactivity and improved cognition after marine oil extract (PCSO-524®) supplementation in children and adolescents with clinical and subclinical symptoms of attention-deficit hyperactivity disorder (ADHD): a randomised, double-blind, placebo-controlled trial

**DOI:** 10.1007/s00213-016-4471-y

**Published:** 2016-12-05

**Authors:** James D. Kean, Jerome Sarris, Andrew Scholey, Richard Silberstein, Luke A. Downey, Con Stough

**Affiliations:** 1 Centre for Human Psychopharmacology, Swinburne University of Technology, PO Box 218 (H24), Hawthorn, VIC 3122 Australia; 2 Department of Psychiatry, Melbourne Clinic, University of Melbourne, Melbourne, Australia

**Keywords:** ADHD, Attention, Hyperactivity, Children, Mood, Cognition, Omega-3, Green-lipped mussel, Marine oil

## Abstract

**Introduction:**

This study investigated the effects of a marine oil extract (PCSO-524**®**) on inattention, hyperactivity, mood and cognition in children and adolescents. PCSO-524**®** is a standardised lipid extract of the New Zealand green-lipped mussel and is an inflammatory modulator that inhibits the 5′-lipoxygenase and cyclooxygenase pathways and decreases concentrations of the pro-inflammatory arachidonic acid (AA).

**Methods:**

PCSO-524**®** or a matched placebo was administered for 14 weeks to 144 participants (123 males/21 females; mean age 8.7 years) with high hyperactivity and inattention in a randomised, double-blind, placebo-controlled study. The primary outcome was the Conners Parent Rating Scale assessing parental reports of behavioural problems. Secondary outcomes assessed changes in cognition and mood.

**Results:**

The results of the present study did not support the hypothesis that PCSO-524® improves parental reports of hyperactivity, inattention and impulsivity in children ages 6 to 14 years over placebo. Repeated measures ANOVA on post hoc subsample analysis indicated significant improvements in hyperactivity (*p =* 0.04), attention (*p* = 0.02), learning (*p =* 0.05) and probability of ADHD (*p =* 0.04) with a medium to large average effect size (*d =* 0.65) in those children who did not meet criteria for combined hyperactivity and inattention. Furthermore, significant improvements in the PCSO-524® group were indicated in a whole sample repeated measures ANCOVA on recognition memory between baseline and week 8 over placebo (*p =* 0.02, *d =* 0.56); this difference was not sustained at week 14.

**Conclusions:**

The results presented indicate that PCSO-524**®** may be beneficial in reducing levels of hyperactivity and inattention in a population of children with clinical and subclinical symptoms of ADHD.

## Introduction

Developmental disorders have detrimental effects on a child’s social, emotional and academic future (Wehmeier et al. 2010). Attention-deficit/hyperactivity disorder (ADHD) is the most prevalent developmental disorder in school-aged children, with ADHD’s prevalence being estimated within Western cultures to be between 5 and 12% (Biederman 2005; Wolraich et al. 2005). While ADHD is diagnosed by the use of criteria that establish clinical levels of hyperactivity, impulsivity and cognitive dysfunction related to inattention and impulsivity (American Psychiatric Association 2000), subclinical levels of these symptoms may still be sufficient to cause significant personal and social distress (Gadit 2003). In fact, subclinical ADHD has demonstrated to be a significant risk factor for alcohol and substance use disorders in later years (Shankman et al. 2009; Malmberg et al. 2011).

Children who present with ADHD symptoms at the subclinical level highlight an area of child mental health that is widespread and largely overlooked. Recent research has indicated a prevalence range for this cohort anywhere from 0.8 to 23% of the population (Balázs and Keresztény 2014). Identifying children with subclinical levels of ADHD may also aid in the broader understanding of gender differences in terms of symptom expression and severity (Rielly et al. 2006) as well as aid in the approach to treatment for those children who are at risk of developing the disorder (Kobor et al. 2012). As such, there is difficulty for parents to have a clear understanding of how to approach their child’s behavioural issues and subsequently find appropriate interventions.

Current pharmacological treatments for ADHD can involve the administration of amphetamine-type and methylphenidate substances, which although efficacious are for many parents an undesirable option. Furthermore, for children who display milder or subclinical levels of inattention or hyperactivity, alternative to amphetamine-type stimulant treatment is arguably preferable. As such, a growing literature concerning alternative treatments proposed for children and adolescents with similar behavioural issues to those with ADHD is developing (Sarris et al. 2011). Although many of these alternative treatments have not been subjected to rigorous scientific clinical trials, there is growing evidence for marine-based intervention efficacy in treating behavioural issues associated with ADHD (Sarris et al. 2011).

One increasingly popular treatment for symptoms of ADHD is supplementation with long-chain polyunsaturated fatty acid (LC PUFAs). Some researchers have argued that these LC PUFAs are a viable alternative to methylphenidate (MPH, e.g. Ritalin®) and other stimulant and non-stimulant pharmaceutical treatments (Richardson 2006; Sinn and Bryan 2007). Longitudinal research into prenatal development has also shown that children who have low levels of docosahexaenoic acid (DHA) in cord blood have increased levels of inattention and hyperactivity at 10 years of age (Kohlboeck et al. 2011). LC PUFAs modify membrane fluidity, neurotransmitter release, cortical connectivity and organisation, as well as decrease levels of inflammatory mediators (Hariri et al. 2012). The involvement of LC PUFAs in processes related to neuronal maturation (Grayson et al. 2014) further suggests that LC PUFA consumption can positively influence these molecular processes and that these changes are also accompanied by behavioural improvements.

A recent clinical trial involving 40 boys with ADHD and 39 healthy controls compared the effect of 16-week administration of combined eicosapentaenoic acid (EPA)/DHA against placebo on behaviour and cognition (Bos et al. 2015). Significant improvements due to the treatment were observed for both the ADHD and control groups due to the EPA/DHA treatment on parent-rated inattention scores but not on the more objectively assessed cognitive processes (Bos et al. 2015). Research findings in this area have been somewhat mixed with several positive findings for LC PUFAs on behavioural outcomes in children and adolescents, as well as several failures to replicate (Sarris et al. 2011). Nevertheless, it should be noted that two recent meta-analyses have suggested positive but small effect sizes for LC PUFAs and behavioural symptoms with children and adolescents with ADHD (Sonuga-Barke et al. 2013; Bloch and Qawasmi 2011). One particular problem in understanding whether omega-3 supplements are efficacious in children with high hyperactivity, inattention and impulsivity is the lack of clarity with regards to the mechanism of action. One promising marine-based preparation that may benefit children and adolescents with behavioural problems and which is rich in LC PUFAs is PCSO-524®, which contains a standardised lipid extract of the New Zealand green-lipped mussel (*Perna canaliculus*). The extract contains a unique combination of free fatty acids, sterol esters, polar lipids and carotenoids that provide a highly condensed form of marine lipids (Kalafatis 1996). Experimental studies demonstrate that the PCSO-524® extract is effective at modulating 5′-lipoxygenase (5-LOX), 12′-lipoxygenase (12-LOX) and cyclooxygenase (COX) pathways, which are responsible for the production of eicosanoids, which include leukotrienes and prostaglandins (Halpern 2000; Whitehouse et al. 1997; Whitehouse and Rainsford 2006). PCSO-524® also elicits an anti-allergic and anti-inflammatory action by controlling the 5-LOX pathway. This regulates both the inflammatory and immune responses, specifically the allergic response induced by interleukin-4 (IL-4) (Dugas 2000). The safety and efficacy of the extract PCSO-524® have been examined in adult, child and adolescent populations, with positive effects in terms of reduced asthmatic (Lello et al. 2012; Emelyanov et al. 2002; Mickleborough et al. 2013), inflammatory bowel disease (Tenikoff et al. 2005) and osteoarthritis symptoms (Zawadzki et al. 2013). With respect to the potential application for symptoms of inattention, impulsivity or hyperactivity, the anti-inflammatory actions of PCSO-524® may benefit children who have clinical or subclinical ADHD by decreasing the ratio of arachidonic acid (pro-inflammatory) to eicosapentaenoic acid (anti-inflammatory) (AA/EPA), which may lead to improvements in associated symptoms (Young et al. 2005; Sorgi et al. 2007).

In the current randomised controlled trial, we investigated the effects of PCSO-524® on the symptoms of hyperactivity, impulsivity, inattention and cognition**.** The primary aim of the current study was to determine if supplementation with PCSO-524® reduced parental reports of hyperactivity and inattention in a population of Australian children and adolescents aged 6 to 14 who had increased levels of hyperactivity or inattention compared with placebo. A secondary aim of the study was to investigate changes in objective computerised measures of cognition and mood as well as electrophysiological measures of brain wave ratios.

## Methods

### Overview

The study was a 14-week randomised, double-blind, placebo-controlled, two-arm, parallel group clinical trial for which children and adolescents were randomised to receive either three capsules (≤45 kg) or four capsules (>45 kg) of PCSO-524® or a matching placebo. This study was approved by the Swinburne University Human Research Ethics Committee (project 2010/175) and was registered with the Australian New Zealand Clinical Trials Registry (ANZCTRN12610000978066). All procedures were conducted in accordance with the Declaration of World Medical Association (2008) and good clinical practice (GCP) guidelines. For an expanded discussion on the methods for this study, see Kean et al. (2013).

### Study aims and hypotheses

The primary aim of the study was to examine the effect of 14 weeks of administration of PCSO-524® on levels of hyperactivity, impulsivity and inattention in children aged 6 to 14 years compared with placebo. The primary outcome was the Conners Parent Rating Scales (CPRS), a comprehensive checklist for acquiring parental reports of the behavioural problems that presented, which was completed every 4 weeks (Conners et al. 1998). Secondary outcomes investigated cognitive changes by the use of the Test of Variables of Attention (TOVA; Greenberg and Waldman 1993) and the Computerised Mental Performance Assessment System (COMPASS; Scholey et al. 2010). Changes in mood were assessed by the use of the Brunel Mood Scale (BRUMS) for adolescents (Terry et al. 1999). These mood scales were completed by the parents independently or (more usually) with the child. The acquisition of resting-state electroencephalography (EEG) was based on previous research into the differentiation of theta/beta and theta/alpha ratios from non-ADHD children (Mann et al. 1992) as well as between subtypes (Clarke et al. 2001). This was conducted in two states: eyes open and eyes closed. For the sake of brevity, the EEG results are reported elsewhere.

### Participants and trial site

One hundred and forty-four children aged between 6 and 14 years were recruited for the study and initially allocated to either a PCSO-524® or placebo group. All cognitive and electrophysiological testing took place within the Swinburne Centre for Human Psychopharmacology at Swinburne University, in Victoria, Australia. Parents completed additional CPRS forms at home during weeks 4, 10 and 18 (4 weeks post-treatment). Families that were based interstate (distant participants) completed all CPRS, BRUMS and symptom checklists in their home environments, at scheduled time points.

### Inclusion criteria

Inclusion criteria were as follows: healthy, non-smoking males and females aged between 6 and 14 years, who had Diagnostic and Statistical Manual of Mental Disorders Fourth Edition (DSM-IV) ADHD rating score of greater than 15, who were fluent in English, had parental or legal guardian consent and verbal consent from the child. The DSM-IV rating scale is a reliable and valid four-point (0 = never or rarely, 1 = sometimes, 2 = often, 3 = very often) 18-item semi-structured interview that assesses symptom severity (Faries et al. 2001). A score of 15 points or higher on the DSM-IV rating scale allowed investigators to establish that participants had elevated levels of hyperactivity, inattention or both. Criteria for ADHD subtypes (inattentive or hyperactive-impulsive), requires six or more scores in the higher range (2 = often, 3 = very often) of the scale for that subtypes (DuPaul et al. 1998).

### Exclusion criteria

Exclusion criteria were as follows: primary medical diagnosis other than ADHD, oppositional defiant disorder or similar behavioural disorders; currently taking any medication (other than stimulants if a formal diagnosis of ADHD or other behavioural disorder has been made); current or history of heart disease, or high blood pressure, or diabetes; health conditions that would affect food metabolism, including the following: food allergies, kidney disease, liver disease and/or gastrointestinal diseases (e.g. irritable bowel syndrome, coeliac disease, peptic ulcers); pregnant or breast feeding; unable to participate in all scheduled visits, treatment plan, tests and other trial procedures according to the protocol; allergy to shellfish; epilepsy or photosensitivity.

### Intervention

The active trial treatment was the naturally occurring omega-3 anti-inflammatory extract PCSO-524®. The lipid extract PCSO-524® of the New Zealand green-lipped mussel is marketed under the brand names Lyprinol® and Omega XL®. The principal ingredients per 260 mg capsule for the active capsules include PCSO-524® GLM pat. lipids (eicosatetraenoic acid)—50 mg (including EPA 7.3 mg and DHA 5.5 mg, natural mono-unsaturated olive oil 100 mg and vitamin E (D-alpha-tocepherol) as an antioxidant 0.225 mg). PCSO-524® also includes sterol esters that consist of mainly myristic acid, palmitic acid, palmitoleic acid, stearic acid, oleic acid and linoleic acid. The sterols found in this fraction included cholesterol, cholesta-3,5-diene, 26,27-dinoergostadienol, cholesta-5,22-dien-3-ol and ergosta-5,22-dien-3-ol. The placebo capsule contained 35.5 mg of olive oil, 112 mg of lecithin, 12 mg of coconut oil and 0.5 mg of 30% beta-carotene. Both treatments were contained within capsules that consisted of gelatin, sorbitol syrup and glycerin. The placebo capsule matched the PCSO-524® capsule in touch, taste, smell and size.

### Randomisation and treatment schedule

Participants were allocated randomly to coded treatment groups. All participants were assigned to treatment groups A or B through the use of a computer-generated random number, which was done by a neutral third party. Blinding was achieved by enlisting a person outside of the project to code the treatments and maintain the key to this code until data collection was completed. An emergency code break envelope was provided to the principal investigator, which was to be opened only in case of emergency. The schedule for testing is presented in Table 1. Weight plays a large role in the digestion and absorption of nutrients, so weight was used to determine how many capsules each child would be required to consume (≤45 kg = 3 capsules; >45 kg = 4 capsules).

**Table 1:** Study outline

	V1	V2		V3		V4	
	W1	W2	W4	W8	W10	W14	W18
On-site participants	Prac	Swin	AH	Swin	AH	Swin	F/Up
Distant participants	Prac	AH	AH	AH	AH	AH	AH
Behavioural and demographic measures							
Structured interview (DSM ADHD rating)	X						
Connors Parent Rating Scale		X	X	X	X	X	X
Global Clinical Impression scale^a^		X		X		X	
Current health and medical questionnaire	X	X	X	X	X	X	
Demographics questionnaire	X						
Omega-3 intake/food diary	X			X			
Cognitive and psychophysiological measures							
COMPASS cognitive battery^a^	X	X		X		X	
Test of Variables of Attention (TOVA)^a^	X	X		X		X	
Brunel Mood Scale (BRUMS)	X	X	X	X	X	X	X
EEG resting state^a^		X		X		X	
Steady-state topography (CPT-AXE)^a^		X		X		X	

^a^These items were not completed by inter-state participants

_*AH* at home, *Swin* at Swinburne, *Prac* practice day, *CPT-AXE* continuous performance task-AXE_

### Procedure

Parents who were interested on behalf of their children contacted the university via telephone or e-mail and underwent a telephone screen to determine the eligibility of their child. Eligible participants who lived interstate were provided with hard copies of consent forms and were enrolled in the study after signed hard copy consent forms had been returned. Eligible children attended the university and completed consent on-site on the practice day. All families underwent a practice day information session or telephone call during which they completed screening questionnaires and familiarised themselves with the study procedures and tests. Local (on-site) participants underwent a baseline session during which they completed all tests (which included an EEG) and were allocated randomly to receive one of the two treatments (PCSO-524®/placebo). They were required to take either three or four capsules daily (in the morning, with breakfast) for 14 weeks. The outcome measures, including the primary outcome, the CPRS, were completed by parents during weeks 2 (baseline), 4, 8, 10, 14 and 18 (Conners et al. 1998).

Families that lived interstate were mailed a study kit that contained the participant’s randomised treatment and were contacted to complete their study forms on specific dates, after their baseline start date. These participants did not complete cognitive assessments. Remaining testing sessions followed the schedule detailed in Table 1. Participants completed a treatment compliance diary and marked each day when their child consumed their treatment.

At the end of the study, participants were required to return the compliance diary as well as any remaining treatment. A follow-up telephone call was undertaken at the conclusion of the study to enquire about compliance rates for each participant. All parents completed a food frequency questionnaire on behalf of the child at the start of the study and then a subsequent 7-day food diary during week 4 to determine if the child’s omega-3 intake from non-trial sources remained consistent throughout the trial. Parents also completed the CPRS and the BRUMS 4 weeks following the cessation of the active or placebo administration (week 18) to determine if there were any changes in mood or behaviour. Parents completed a symptom checklist to monitor for any adverse events. Parents were questioned about any non-specific adverse events (AEs) at each scheduled time point (weeks 4, 8, 10, 14 and 18).

### Outcome measures

The a priori primary outcome was the CPRS (Conners et al. 1998). Secondary outcomes included the computerised COMPASS cognitive battery, designed to allow assessment across the major cognitive domains, i.e. attention, working memory, secondary memory and executive function (Scholey et al. 2010). The following cognitive COMPASS tasks were administered: word presentation, immediate word recall, picture presentation, simple reaction time, choice reaction time, numeric working memory, delayed word recall, delayed word recognition and delayed picture recognition. Each outcome was scored in terms of accuracy and speed of response. The TOVA was employed to objectively assess symptoms of inattention and impulsivity. The TOVA is a computer-based assessment of inattention (Greenberg and Waldman 1993). It is considered to be a gold standard test for ADHD and associated symptoms (Llorente et al. 2008). The test has two modes divided into four quarters. The *target infrequent* mode in quarters 1 and 2 (36 targets; 126 non-targets) is the traditional form used to measure vigilance. This is followed by the *target frequent* mode in quarters 3 and 4 (126 targets; 36 non-targets) that denoted the high inhibition demand mode. The BRUMS*,* known formerly as the Profile of Mood States-Adolescents (POMS-A), was used to assess the mood states of the children. The BRUMS contained 24 simple mood descriptors such as angry, energetic, nervous and unhappy. It was designed specifically for adolescent populations, and the validation process can be found in Terry et al. (1999).

The Global Clinical Impression (GCI) scale was used to measure illness severity, global improvement or change and therapeutic response. The GCI is rated on a seven-point scale, with the severity of illness scale using a range of responses from one (normal) through to seven (among the most severely ill patients (Busner and Targum 2007)). Parents also completed a Food Frequency Questionnaire (FFQ) that provided a detailed description of the child’s food intake. During week 4 of the trial, parents completed a 7-day food diary that recorded what the child ate for breakfast, lunch and dinner, as well as any significant snacks throughout those days. This diary was compared with the FFQ at baseline to determine any changes in diet that may have occurred during the study. Resting EEG and steady-state topography (SST) were recorded in on-site participants; however, the results due to their size are not reported here.

### Statistical analysis

Analysis was performed using the Statistical Package for the Social Sciences (SPSS v.20). Repeated measures analysis of variance (ANOVA) were applied to all data, and in each analysis, *diagnosis* and *medication* were included as categorical covariates. Analysis of covariance (ANCOVA) was applied to all cognitive data to control for any confounding variables that may have impacted the performance on the measures. A multiple comparison test (Bonferroni) was applied to all data, which included all exploratory subsample analyses and the effect sizes (Cohen’s *d*) provided. Cohen’s effect sizes are interpreted as 0.2 = small, 0.5 = medium and 0.8 = a large effect size. Significance level was set at a *p <* 0.05. Intention-to-treat analysis, which used the last observation carried forward, was applied to any missing data from endpoint analysis, which included any eligible data from participants who dropped out.

## Results

### Participant characteristics

Three hundred and fifty-one participants were screened initially for the trial, which resulted in 144 participants who met inclusion criteria and who were randomised to receive either PCSO-524® or a placebo for 14 weeks. The mean age was 8.7 years (SD = 2.24) of whom 123 were male and 21 were female (see Table 2 for demographic data). In total, the data of 112 participants were included for final analysis through the use of intention-to-treat analysis, with the final observation carried forward. Following random assignment and dropouts, there was a significant demographic difference in the number of females in the PCSO-524® group compared to the placebo group (*N* = 17; PCSO-524® *n* = 14). To further explore this, a repeated measure analysis was conducted investigating the effect of treatment on male and females only. This data is reported further on.

**Table 2:** Demographic characteristics of study participants (*N* = 112)

		Mean	SD	Min	Max
Age	yrs	8.82	2.27	6	14
Height	cm	138.45	14.64	100	172
Weight	kg	35.75	13.8	19	90
Education	yrs	4.65	2.35	1	10
Handedness *n* (%)	R	99 (88.4%)			
	L	11 (9.8%)			
	L/R	2 (1.7%)			

*yrs* years, *n* number, *cm* centimetre, *kg* kilogram, *R* right handed, *L* left handed, *L/R* left and right handed

Whole sample treatment group differences were noted in secondary outcome, the COMPASS cognitive task, which included significant improvements in working memory on three domains in favour of PCSO-524®. No further treatment group differences were found in an analysis of the entire sample on remaining primary and secondary outcomes.

**Fig. 1:**
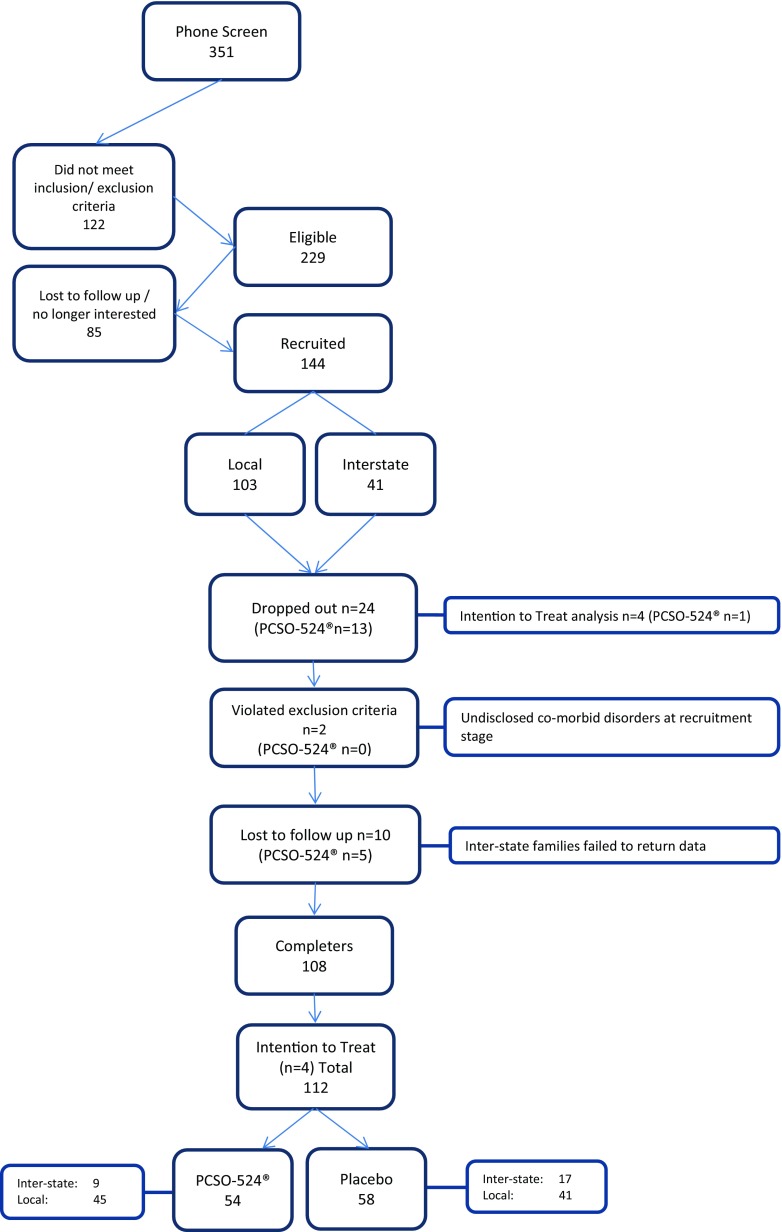
Flowchart of study protocol

As the sample population included children taking pharmaceutical medications, careful interpretation of the analysis and outcomes was needed. Each result was analysed using diagnosis and medication status as a covariate. Any statistical influence of these covariates on any outcome is noted in the relevant analysis. These participant numbers are broken down by groups including on-site testing, distant testing and gender as well as to which treatment group these participants were randomised (see Table 3 and Fig. 1).

**Table 3:** Full sample demographics broken down by location, sex, diagnosis and medication

	Overall (*N* = 144)		Total distant participants (*N* = 41)		Total on-site participants (*N* = 103)		Final sample (on-site) (*N* = 112)	
	PCSO-524®	Placebo	PCSO-524®	Placebo	PCSO-524®	Placebo	PCSO-524® (45)	Placebo (41)
Male	57	66	12	24	44	43	40	55
Female	17	4	4	1	13	3	14^a^	3
Diagnosis	36	37	8	18	28	19	22	28
Medication^b^	23	29	7	14	16	15	13	22
					Ritalin	LA (mg) SA (mg)	4 (20) 0 (0)	2 (30) 5 (9)
					Concerta	LA (mg) SA (mg)	7 (36) 0 (0)	9 (35) 0 (0)
					Dexam	LA (mg) SA (mg)	1 (12.5) 0 (0)	0 (0) 2 (10)
					Strattera	LA (mg) SA (mg)	1 (40) 0 (0)	2 (32) 0 (0)

*Dexam* dexamphetamine, *LA* long acting, *mg* average milligram per dose of that specific medication, *SA* short acting

^a^There was a significant difference in the number of females between treatment groups

^b^Eight participants failed to provide dosage (mg) information (PCSO-524® *n* = 2); two could not provide medication type (PCSO-524® *n* = 0)

### ADHD assessment scales

#### Conners Parent Rating Scale

A baseline summary of the CPRS data was included investigating differences between demographic data (see Table 4). Comparisons between gender, medication status, diagnosis status and testing location were conducted using paired sample *t* test. Any significant outcome was further investigated using Wilcoxon signed-rank test for non-parametric datasets. There were significant baseline differences for the peer relations (*p <* 0.05), oppositional defiant disorder (*p <* 0.01), impaired relationships (*p <* 0.01) and impaired home life (*p <* 0.01) outcomes between the diagnosed and non-diagnosed groups as well as significant differences on the peer relations (*p <* 0.05) and impaired relationships (*p <* 0.05) between medicated and non-medicated groups. No further differences were noted in any other demographic data at baseline. Despite these demographic differences, following randomisation, there were no significant differences between treatment groups on any of the CPRS outcomes.

**Table 4:** Baseline scores for Conners Parent Rating Scale based on demographic data (*N* = 112)

	Sex		Medicated		Diagnosed		On-site	
	Male	Female	YES	No	Yes	No	Yes	No
Inattention	78.84 ± 11.09	83.25 ± 10.21	81.25 ± 8.79	79.33 ± 11.65	81.27 ± 8.84	78.92 ± 12.10	80.50 ± 10.84	78.00 ± 10.97
Hyperactivity	80.85 ± 12.25	80.75 ± 15.34	85.09 ± 7.64	80.07 ± 13.37	84.49 ± 9.26	79.46 ± 13.53	82.20 ± 12.48	79.54 ± 11.03
Learning problems	68.66 ± 14.87	73.00 ± 16.28	73.69 ± 13.14	67.50 ± 15.20	70.47 ± 14.45	68.52 ± 15.16	69.33 ± 15.49	69.35 ± 12.79
Executive function	69.12 ± 11.76	74.38 ± 16.61	71.56 ± 10.80	70.64 ± 13.38	71.56 ± 11.76	70.46 ± 13.29	71.70 ± 13.04	68.46 ± 11.10
Aggression	77.36 ± 14.67	69.88 ± 17.98	79.41 ± 12.86	75.78 ± 15.71	79.69 ± 13.17	74.83 ± 15.91	76.66 ± 15.33	77.46 ± 13.98
Peer relations	70.78 ± 16.78	63.38 ± 22.36	**78.81 ± 15.10**	67.75 ± 17.16	**77.44 ± 15.36**	66.44 ± 17.21	71.01 ± 17.41	71.08 ± 17.16
DSM inattention	75.45 ± 10.74	79.50 ± 14.16	77.25 ± 9.25	76.74 ± 11.97	77.24 ± 9.72	76.63 ± 12.20	77.70 ± 11.27	74.35 ± 10.75
DSM hyperactivity	79.14 ± 12.38	78.75 ± 15.56	83.31 ± 8.93	78.37 ± 13.29	82.78 ± 10.38	77.73 ± 13.23	80.78 ± 12.54	76.85 ± 11.37
Conduct disorder	70.07 ± 15.61	70.00 ± 16.41	73.06 ± 15.10	69.57 ± 15.86	73.29 ± 15.07	68.68 ± 15.89	70.70 ± 15.83	70.31 ± 15.37
Oppositional defiant disorder	75.88 ± 12.81	66.00 ± 17.62	79.16 ± 10.98	73.64 ± 13.95	**79.24 ± 11.27**	72.44 ± 14.04	75.02 ± 13.76	76.08 ± 12.09
Global ADHD index	79.88 ± 11.52	80.38 ± 11.70	84.53 ± 7.26	78.82 ± 12.21	84.04 ± 7.48	77.98 ± 12.78	80.85 ± 11.57	79.42 ± 10.34
Impaired school life	2.21 ± 0.93	2.25 ± 0.89	2.28 ± 0.92	2.20 ± 0.91	2.24 ± 0.96	2.21 ± 0.88	2.24 ± 0.90	2.15 ± 0.97
Impaired relationships	1.70 ± 0.98	1.00 ± 1.41	**1.97 ± 1.03**	1.51 ± 1.04	**2.00 ± 1.00**	1.40 ± 1.02	1.67 ± 1.02	1.58 ± 1.17
Impaired home life	1.99 ± 0.96	1.38 ± 1.19	2.31 ± 0.86	1.80 ± 1.03	**2.33 ± 0.83**	1.68 ± 1.04	1.98 ± 1.03	1.88 ± 0.95
ADHD probability	88.01 ± 20.74	87.50 ± 15.75	94.75 ± 8.26	86.41 ± 22.43	93.67 ± 14.53	85.46 ± 22.13	88.98 ± 19.73	88.58 ± 19.89

Significant differences are in bold

Analysis of the primary outcome found no significant differences between treatment groups on CPRS following 14-week supplementation. To further understand potential treatment effects, we also conducted post hoc analyses with subgroups. This was done to better understand whether there was a treatment effect related to the severity of the symptoms displayed by children and adolescents. Subsample analysis indicated significant treatment effects for participants who had less-severe symptoms (high inattention, high hyperactivity or no subtype—non-combined type) than those who had more severe symptoms (combined high hyperactivity and inattention—combined type) based on attention-deficit/hyperactivity disorder IV rating scale scores (DuPaul et al. 1998).

Non-combined type (*NCT*) (*N* = 43; PCSO-524® *n* = 23) analysis revealed a significant improvement in children who consumed PCSO-524® on CPRS scores of hyperactivity, learning abilities and improved behaviour at home, as well as improvements on DSM scores of attention and hyperactivity. Furthermore, there was a significant reduction in ADHD probability ratings in children who consumed PSCO-524® compared with placebo; this result was associated with a main effect for diagnosis (*p =* 0.03) (see Table 5). No baseline differences were seen in the NCT sample. There were also no significant differences between treatment groups in terms of those with high inattention (*p =* 0.08) or high hyperactivity (*p =* 0.10) at baseline. The NCT subsample was comprised of three classifications, which included those who met criteria for high inattention, those who met criteria for high hyperactivity and those who did not meet criteria for either high inattention or high hyperactivity, denoted from here as non-subtype group. Further analysis of these groups found that those children taking PCSO-524® and displaying high inattention (*N* = 19; PCSO-524® *n* = 11) improved in parental ratings of executive function (*p =* 0.01; *d =* 0.38), aggression (*p =* 0.01; *d =* 0.70), conduct (*p <* 0.01; *d =* 1.03) and oppositional defiance (*p =* 0.04; *d =* 0.46). Improvements in aggression and oppositional defiance were associated with main effects for medication (*p <* 0.05) and diagnosis (*p <* 0.05). This may indicate the potential for PCSO-524® as an adjunct therapy in ADHD-diagnosed children displaying specific issues with attention; however, future studies are needed to establish this association. Improvements in executive function were also associated with a main effect for diagnosis (*p <* 0.05). There were no improvements on any domain for those children displaying symptoms of high hyperactivity (*N* = 10; PCSO-524® *n* = 4) or the non-subtype group (*N* = 15, PCSO-524® *n* = 9). Only a single baseline difference was noted in the conduct issues outcome for the non-subtype group (*p <* 0.05).

**Table 5:** Non-combined-type CPRS scores

Non-combined type							
		PCSO-524 (*n* = 23)		Placebo (*n* = 20)			
Variables	*n*	Baseline	Week 14	Baseline	Week 14	*p* value^a^	Cohens *d*
Inattention	43	74.57 ± 12.17	61.61 ± 12.45	74.10 ± 11.49	67.55 ± 12.14	0.11	0.48
Hyperactivity	43	73.04 ± 14.52	62.87 ± 14.24	75.00 ± 13.58	71.70 ± 13.08	0.04	0.64
Learning problems	43	63.48 ± 17.69	57.61 ± 12.88	68.90 ± 12.62	66.15 ± 14.79	0.05	0.62
Executive function	43	65.30 ± 13.29	57.09 ± 11.81	65.45 ± 12.64	63.20 ± 11.29	0.09	0.53
Aggression	43	72.96 ± 16.56	65.78 ± 17.73	68.75 ± 15.05	69.20 ± 15.50	0.49^c^	0.20
Peer relations	43	64.87 ± 15.94	61.65 ± 18.24	64.45 ± 18.73	60.80 ± 14.23	0.85^c^	0.05
DSM inattention	43	71.83 ± 12.58	58.00 ± 11.84	70.80 ± 12.56	66.75±13.10	0.02	0.70
DSM hyperactivity	43	71.04 ± 13.86	62.39 ± 12.54	72.30 ± 12.63	70.00 ± 11.81	0.04	0.62
Conduct disorder	43	65.39 ± 14.68	56.83 ± 14.32	64.00 ± 13.61	63.90 ± 15.58	0.12	0.47
Oppositional defiant disorder	43	68.00 ± 13.76	62.00 ± 14.54	66.90 ± 12.98	62.50 ± 10.45	0.89	0.04
Global ADHD index	43	73.87 ± 11.97	63.13 ± 11.86	73.05 ± 11.50	68.35 ± 11.36	0.14^c^	0.45
Impaired school life	43	1.83 ± 0.89	1.26 ± 1.01	2.05 ± 1.00	1.75 ± 1.07	0.13	0.47
Impaired relationships	43	1.09 ± 1.00	1.09 ± 1.08	1.30 ± 0.98	1.35 ± 0.88	0.41^b^	0.27
Impaired home life	43	1.39 ± 0.99	0.87 ± 1.01	1.50 ± 1.15	1.55 ± 0.83	0.02	0.73
ADHD probability	43	79.61 ± 24.93	51.30 ± 28.44	81.75 ± 18.36	68.70 ± 29.82	0.04^c^	0.60

^a^Significant values—Bonferroni multiple comparison test

^b^Outcome was associated with a main effect for medication

^c^Outcome was associated with a main effect for diagnosis

Group analysis of the combined type (*CT*) (*N* = 65; PCSO-524® *n* = 29) revealed an improvement in attention, executive function and DSM ratings of attention in the placebo group (see Table 6); however, both symptoms of executive function and DSM ratings of attention showed a significant main effect for medication status, which highlights the possible influence of pharmaceutical treatment on parental ratings of participant’s behaviour. No baseline differences were noted in the CT sample.

**Table 6:** Combined-type CPRS scores

Combined type							
		PCSO-524 (*n* = 29)		Placebo (*n* = 36)			
Variables	*n*	Baseline	Week 14	Baseline	Week 14	*p* value^a^	Cohens *d*
Inattention	65	83.59 ± 9.19	77.34 ± 13.23	83.56 ± 8.14	71.03 ± 13.61	0.04	0.47
Hyperactivity	65	87.03 ± 6.34	78.10 ± 14.23	86.22 ± 8.17	74.97 ± 14.45	0.31	0.22
Learning problems	65	70.28 ± 15.21	68.90 ± 15.78	72.56 ± 13.03	64.81 ± 14.76	0.17	0.27
Executive function	65	75.34 ± 12.25	73.00 ± 13.69	73.97 ± 10.30	63.56 ± 11.64	0.00^b^	0.75
Aggression	65	79.83 ± 13.07	73.66 ± 15.18	81.44 ± 13.25	68.39 ± 17.60	0.17	0.32
Peer relations	65	74.48 ± 18.10	72.14 ± 16.06	75.83 ± 14.70	68.89 ± 18.82	0.26	0.18
DSM inattention	65	80.83 ± 9.32	75.97 ± 12.74	80.33 ± 8.08	67.44 ± 13.08	0.01^b^	0.66
DSM hyperactivity	65	85.90 ± 6.60	77.28 ± 14.59	84.75 ± 9.17	73.00 ± 14.73	0.19	0.29
Conduct disorder	65	74.86 ± 15.71	66.07 ± 14.43	74.17 ± 15.65	62.56 ± 17.16	0.23	0.22
Oppositional defiant disorder	65	79.45 ± 10.67	69.41 ± 18.67	81.22 ± 10.78	67.92 ± 15.51	0.54^b^	0.09
Global ADHD index	65	85.03 ± 7.70	78.14 ± 13.47	85.25 ± 8.78	71.86 ± 14.01	0.04	0.46
Impaired school life	65	2.17 ± 0.93	1.93 ± 1.07	2.61 ± 0.73	1.75 ± 1.05	0.30	0.17
Impaired relationships	65	1.76 ± 1.09	1.79 ± 0.90	2.11 ± 0.89	1.56 ± 1.08	0.17	0.24
Impaired home life	65	2.21 ± 0.94	1.86 ± 0.74	2.36 ± 0.72	1.58 ± 1.05	0.06^b^	0.30
ADHD probability	65	93.69 ± 16.71	80.79 ± 29.61	94.89 ± 15.64	72.97 ± 28.58	0.18	0.27

^a^Significant values—Bonferroni multiple comparison test

^b^Outcome was associated with a main effect for medication

#### Clinical Global Impression scale

There were no significant differences between treatment groups at baseline or during the following 14 weeks of treatment.

### Cognitive assessment

#### Computerised Mental Performance Assessment System

**Fig. 2:**
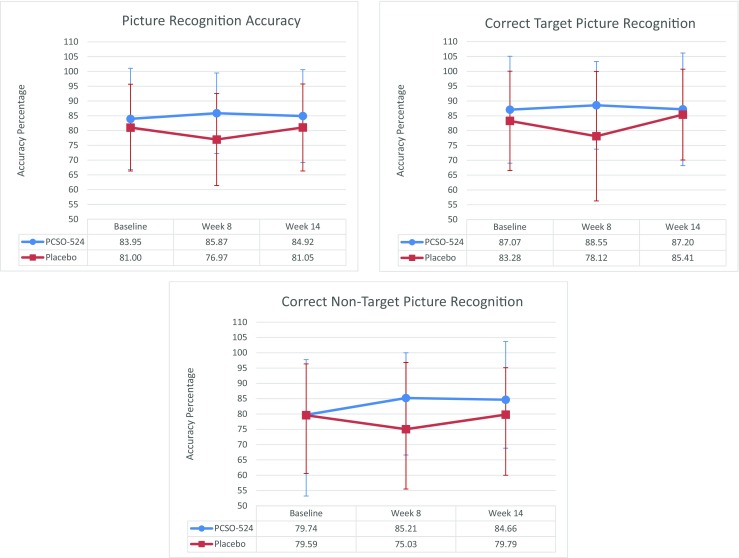
Graphs showing accuracy percentage on picture recognition accuracy, accuracy for correct target picture recognition and accuracy for correct non-target picture recognition

There were no significant differences between groups at baseline for any of the cognitive measures. Results of the reaction time cognitive data did not include *fast guesses* (<200 ms). Whole sample analysis (*N* = 85) revealed significant effects in favour of PCSO-524® between baseline and week 8. ANCOVA analysis was conducted using diagnosed and medication status as well as baseline scores as covariates. The main outcomes were improved memory accuracy scores of participants in the PCSO-524® group when they recalled target (*p =* 0.05, *d =* 0.48) and non-target (*p =* 0.02, *d =* 0.56) pictures correctly; this is complemented by significant overall picture recognition accuracy (*p =* 0.02, *d =* 0.56) (see Fig. 2a–c). There were no main effects for diagnosis or medication on any of the whole sample outcomes.

#### COMPASS subsample analysis

Baseline differences (*p* < 0.05) were noted between treatment groups on three subscores for the *word recognition* task in the diagnosed (*Ds*) (*n* = 28; PCSO-524® *n* = 14) subsample only. A single significant difference was noted on the delayed word recall task in the non-diagnosed (*NDs*) (*n* = 57; PCSO-524® *n* = 31) subsample (*p <* 0.05). No other baseline differences were noted in either subsample. There were no main effects for medication in the diagnosed subsample on any COMPASS outcome.

**Fig. 3:**
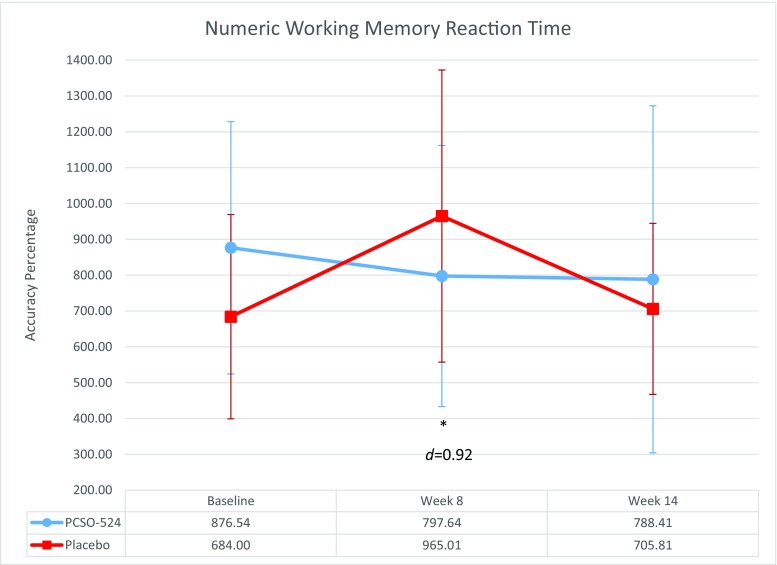
Effects of PCSO-524® (Lyprinol/Omega-XL) on the numeric working memory outcomes. Graphs depict mean COMPASS reaction time scores with SD at baseline, week 8 and week 14 compared to placebo. Significant differences (*p < 0.05) are indicated; Cohen’s *d* effect sizes are provided

In the NDs subsample, significant improvements were seen on the same working memory scores as in the whole sample analysis. ANCOVA analysis of the subsample revealed that those in the PCSO-524® group had improved picture recognition (*p =* 0.03, *d =* 0.67), correct target picture recognition (*p* = 0.05, *d =* 0.58) and correct non-target picture recognition (*p* = 0.02, *d* = 0.69). In the *Ds* subsample, a significant improvement in numeric working memory (Fig. 3) was demonstrated in those taking PCSO-524® between baseline and week 8 (*p =* 0.02, *d* = 0.92).

#### Test of Variables of Attention

The TOVA is a highly demanding task that requires significant sustained attention during a long period of time. Analysis was conducted using a 2 × 2 repeated measures ANOVA (quarter × time) on each mode (target-infrequent [first half] mode or target-frequent [second half]) mode to evaluate the individual influences of each quarter on the data. Any quarter that had excessive omission errors (>90%) was considered invalid. Participants whose data was deemed invalid were removed from the final analysis. Seventy-seven participant datasets were included in the final analysis. There were no significant differences between groups at baseline. No statistically significant differences were found between the treatment groups in whole sample analysis.

#### TOVA subsample analysis

In the NDs subsample (*n* = 52; PCSO-524® *n* = 28), the PCSO-524® group showed significantly increased speed when they gave a correct response to a target following an error (post-commission) and demonstrated sustained consistency of those responses compared with placebo over the 14 weeks during the target-infrequent mode (see Table 7). In the target-frequent mode, those in the placebo group made significantly more multiple responses than those in the PCSO-524® group (see Table 8). This demonstrated an example of improved inhibitory mechanisms. For the Ds sample (*n* = 25; PCSO-524® *n* = 12), those in the PCSO-524® group showed slower reaction times when they responded to target stimuli (*p =* 0.09, *d =* 0.18), which was complemented by another trend, which demonstrated that those children who consumed PCSO-524® made fewer errors than the placebo group over 14 weeks (*p =* 0.08, *d =* 0.20).

**Table 7:** Test of Variables of Attention (TOVA) target-infrequent means and standard deviations for non-diagnosed subsample

		First half (target infrequent—attention)					
		Baseline		Week 14		*p* value^a^	Cohen’s *d*
	*n*	QTR 1	QTR 2	QTR 1	QTR 2		
Response time variability							
PCSO-524®	38	235.14 ± 97.90	237.69 ± 105.96	203.83 ± 69.07	239.73 ± 90.72	0.19	0.47
Placebo		236.32 ± 89.98	209.14 ± 97.54	287.52 ± 123.32	238.60 ± 86.55		
Response time							
PCSO-524®	37	632.32 ± 129.48	655.52 ± 123.68	595.83 ± 133.68	687.32 ± 164.01	0.83	0.10
Placebo		632.00 ± 136.38	663.14 ± 160.27	650.35 ± 120.80	648.00 ± 132.14		
*D*′ prime							
PCSO-524®	38	2.30 ± 1.73	2.42 ± 1.66	2.00 ± 1.51	2.31 ± 2.10	0.63	0.04
Placebo		2.14 ± 1.38	2.64 ± 1.63	2.27 ± 1.96	2.09 ± 1.88		
Correct response							
PCSO-524®	38	23.04 ± 10.74	22.77 ± 11.13	24.61 ± 8.49	22.09 ± 9.51	0.96	0.13
Placebo		25.18 ± 8.96	23.57 ± 8.89	23.25 ± 10.28	20.15 ± 10.12		
Correct non-response							
PCSO-524®	38	106.39 ± 25.85	115.15 ± 15.53	102.65 ± 31.43	109.41 ± 28.31	0.71	0.10
Placebo		109.27 ± 14.52	115.86 ± 12.16	108.10 ± 17.63	108.05 ± 17.85		
Errors of omission							
PCSO-524®	36	0.33 ± 0.27	0.36 ± 0.31	0.31 ± 0.23	0.39 ± 0.26	0.82	0.02
Placebo		0.30 ± 0.24	0.34 ± 0.25	0.34 ± 0.28	0.39 ± 0.29		
Errors of commission							
PCSO-524®	36	0.11 ± 0.13	0.07 ± 0.10	0.18 ± 0.24	0.12 ± 0.22	0.46	0.19
Placebo		0.13 ± 0.12	0.07 ± 0.08	0.12 ± 0.11	0.10 ± 0.12		
Post commission							
PCSO-524®	38	5.11 ± 3.80	4.15 ± 3.56	6.70 ± 5.51	4.95 ± 5.27	0.89	0.01
Placebo		7.86 ± 5.01	4.76 ± 4.10	6.60 ± 3.78	4.95 ± 3.79		
Post commission response time							
PCSO-524®	38	627.29 ± 252.50	626.23 ± 269.37	555.00 ± 209.43	575.41 ± 265.83	0.16	0.43
Placebo		602.00 ± 126.53	530.95 ± 255.98	705.90 ± 232.93	614.60 ± 221.22		
Post commission variability							
PCSO-524®	38	179.18 ± 156.39	145.77 ± 137.25	127.13 ± 94.86	157.59 ± 169.81	0.02	0.66
Placebo		200.45 ± 136.85	125.14 ± 131.84	268.30 ± 163.49	201.20 ± 145.11		
Anticipatory response							
PCSO-524®	37	0.03 ± 0.07	0.02 ± 0.03	0.05 ± 0.13	0.05 ± 0.13	0.55	0.18
Placebo		0.02 ± 0.02	0.01 ± 0.02	0.03 ± 0.06	0.03 ± 0.04		
Multiple responses							
PCSO-524®	38	3.22 ± 5.56	2.42 ± 4.66	2.65 ± 3.61	2.77 ± 4.54	0.54	0.25
Placebo		1.36 ± 2.48	1.76 ± 3.74	3.90 ± 6.78	4.55 ± 5.96		

^a^Bonferroni multiple comparison test

**Table 8:** Test of Variables of Attention (TOVA) target-frequent means and standard deviations for non-diagnosed subsample

		Second half (target frequent—inhibition)					
		Baseline		Week 14		*p* value^a^	Cohen’s *d*
	*n*	QTR 3	QTR 4	QTR 3	QTR 4		
Response time variability							
PCSO-524®	24	250.55 ± 94.20	262.05 ± 108.14	241.25 ± 57	254.42 ± 85.89	0.30	0.45
Placebo		264.60 ± 122.51	270.56 ± 136.35	283.75 ± 103.90	294.79 ± 100.07		
Response time							
PCSO-524®	25	547.14 ± 123.16	540.50 ± 161.68	586.48 ± 145.97	589.37 ± 152.46	0.65	0.14
Placebo		541.40 ± 133.95	531.24 ± 150.48	582.00 ± 171.18	551.71 ± 133.53		
*D*′ prime							
PCSO-524®	24	1.49 ± 1.18	1.11 ± 0.92	1.76 ± 1.74	1.29 ± 1.62	0.49	0.23
Placebo		1.24 ± 1.21	1.22 ± 1.10	1.24 ± 1.29	1.12 ± 0.97		
Correct response							
PCSO-524®	25	87.48 ± 34.20	83.10 ± 34.48	78.10 ± 34.93	68.11 ± 35.87	0.56	0.03
Placebo		84.70 ± 35.34	82.18 ± 36.44	72.69 ± 33.95	71.57 ± 33.43		
Correct non-response							
PCSO-524®	25	23.33 ± 8.60	19.95 ± 8.65	26.52 ± 8.35	26.00 ± 8.75	0.70	0.12
Placebo		21.70 ± 7.00	20.88 ± 7.94	24.69 ± 6.73	25.88 ± 7.82		
Errors of omission							
PCSO-524®	21	0.27 ± 0.27	0.29 ± 0.27	0.34 ± 0.27	0.41 ± 0.29	0.99	0.03
Placebo		0.28 ± 0.21	0.32 ± 0.26	0.37 ± 0.26	0.36 ± 0.25		
Errors of commission							
PCSO-524®	21	0.34 ± 0.23	0.41 ± 0.22	0.27 ± 0.22	0.28 ± 0.23	0.63	0.11
Placebo		0.36 ± 0.17	0.37 ± 0.19	0.28 ± 0.17	0.31 ± 0.18		
Post commission							
PCSO-524®	24	10.29 ± 5.84	11.45 ± 5.32	7.15 ± 4.85	7.22 ± 8.67	0.26	0.30
Placebo		11.00 ± 5.14	10.65 ± 5.43	8.88 ± 4.75	8.43 ± 5.14		
Post commission response time							
PCSO-524®	24	545.76 ± 146.87	541.00 ± 178.39	587.30 ± 171.72	549.39 ± 190.90	0.99	0.09
Placebo		588.65 ± 220.08	532.76 ± 125.79	638.31 ± 233.62	533.07 ± 185.12		
Post commission variability							
PCSO-524®	24	189.90 ± 132.14	217.10 ± 132.14	217.35 ± 154.35	267.00 ± 144.74	0.84	0.13
Placebo		235.35 ± 164.26	283.24 ± 145.12	240.38 ± 171.10	203.21 ± 144.98		
Anticipatory response							
PCSO-524®	24	0.05 ± 0.10	0.07 ± 0.12	0.06 ± 0.14	0.07 ± 0.14	0.86	0.06
Placebo		0.06 ± 0.12	0.09 ± 0.18	0.05 ± 0.06	0.06 ± 0.08		
Multiple responses							
PCSO-524®	24	3.33 ± 5.35	6.70 ± 6.07	2.90 ± 4.91	4.79 ± 6.17	0.05	0.48
Placebo		5.85 ± 7.13	7.00 ± 7.35	7.19 ± 6.59	6.21 ± 6.42		

^a^Bonferroni multiple comparison test

### Mood assessment

#### Brunel Mood Scales (BRUMS)

The data of 108 participants were used for analysis. There were no significant differences between treatment groups at baseline. Whole sample analysis revealed significant changes in feelings of fatigue and confusion. Fatigue increased in the PCSO-524® group until week 8 (*p* = 0.02, *d* = 0.49), when it levelled off at week 14 (*p* = 0.01, *d* = 0.53), whereas there was a consistent decrease in fatigue in those children in the placebo group. Both treatment groups had reduced feelings of confusion during the 14 weeks; however, the placebo group did so more significantly at week 8 (*p* = 0.02, *d* = 0.46) and at week 14 (*p* = 0.01, *d* = 0.55).

#### BRUMS subsample analysis

In the Ds subsample, there was a significant decrease in feelings of confusion in the placebo group at week 8 (*p =* 0.00, *d =* 1.14) and at week 14 (*p =* 0.03, *d =* 0.68). Subsample analysis of the NDs subsample demonstrated a significant reduction in feelings of fatigue in the placebo group between baseline and week 14 (*p =* 0.02, *d =* 0.63), compared with PCSO-524®. This was accompanied by a trend in decreased feelings of depression (*p =* 0.07, *d =* 0.48) and confusion (*p =* 0.07, *d =* 0.47) in both treatment groups, with greater significance in the placebo group.

### Sex differences

To evaluate the difference between gender on behavioural outcomes, a repeated measure ANOVA was conducted on males in the study. An analysis on females was not conducted due to the low number of females at the conclusion of the study coupled with the difference in numbers between groups. A repeated measures ANOVA using Bonferroni multiple comparison analysis was conducted on the male-only cohort. There were no significant differences between treatment groups on any CPRS domains following a whole sample analysis (*N* = 91; PCSO-524® *n* = 38) or for a CT analysis (*N* = 56; PCSO-524® *n* = 21). In an NCT analysis of males only (*N* = 35; PCSO-524® *n* = 17), there were significant improvements in CPRS ratings of attention (*p =* 0.03, *d =* 0.74), hyperactivity (*p =* 0.00, *d =* 1.13), learning problems (*p =* 0.03, *d =* 0.78), as well as DSM ratings of attention (*p =* 0.02, *d =* 0.80) and hyperactivity (*p =* 0.00, *d =* 1.09). There were further improvements on the global index of ADHD (*p =* 0.01, *d =* 0.86) and ratings of impaired home life (*p =* 0.01, *d =* 0.82), as well as improved ADHD probability ratings (*p =* 0.00, *d =* 0.91). The CPRS ratings of attention and the ADHD probability scores were associated with main effects for diagnosis and medication status.

### Adverse effects

There was a significant difference between groups in ‘feeling colder’ at baseline; however, this difference dissipated at the end of the study. No other differences in symptoms were noted at baseline or during the course of the study (see Table 9).

**Table 9:** Symptom checklist outcomes at baseline and at week 14 between treatment groups

Symptom	Baseline differences	Week 14
Change in energy	0.66	0.84
Skin irritation	0.13	0.69
Feel colder	0.01^a^	0.48
Feeling hotter	0.08	0.14
Feel dizzy	0.19	0.54
Sweating	0.53	0.44
Blurred vision	0.26	0.46
Nauseous	0.63	0.87
Heart rate increased	0.89	0.86
Dry mouth	0.34	0.78
Stomach pains	0.66	0.58
Eye pains	0.50	0.81
Ear pains	0.32	0.60
Change in bowel patterns	0.90	0.62
Bruising	0.12	0.69
Change in breathing	0.73	0.40
Change in hunger	0.34	0.94
Change in thirst	0.71	0.38
Constipation	0.62	0.60
Urination	0.37	0.23
Fatigue	0.44	0.86
Stress	1.00	0.44
Anxiety	0.83	0.22
Mood	0.08	0.06
Memory	0.90	0.56
Attention	0.73	0.68
Sleep patterns	0.07	1.00
Tremors	0.13	0.06^b^

^a^Significant baseline difference in symptoms of *feeling colder* which disappeared at study’s end

^b^Trending significance for feelings of *tremors* with reductions in the PCSO-524® group and increases in the placebo group

### Post-treatment follow-up

Week 18 final behaviour (CPRS) and mood (BRUMS) follow-up compliance completion was low (53%). Paired sample *t* test indicated a significant worsening in executive function for the placebo group in the CT condition (*p =* 0.05; *d = −*0.34). There was a significant increase in tension (*p =* 0.05, *d* = 0.36) throughout the whole sample in the PCSO-524® group, following cessation of treatment. In subsample analysis, those children who had been diagnosed with ADHD and who had finished taking PCSO-524® showed increased tension (*p =* 0.05, *d* = 0.51) and symptoms of depression (*p =* 0.02, *d* = 0.58). These results should be interpreted with caution due to the low completion rate.

### Dropouts, withdrawals and compliance

There were 24 participant dropouts. The majority of dropouts occurred due to family issues (*n* = 9 placebo; PCSO-524® *n* = 4) that left the participant unable to complete testing procedures (see Fig. 1 for a study recruitment flowchart). A common issue with younger participants was an inability to swallow the capsules (placebo *n* = 7; PCSO-524® *n* = 4). Some participants began ADHD medication or made adjustments to their medication regimen (placebo *n* = 3; PCSO-524® *n* = 2) and were excluded from further testing. Two participants withdrew from the study due to symptoms; one participant complained of an increase in noise sensitivity (placebo), and one parent reported an increase in the child’s hyperactivity while at home (PCSO-524®). Two further participants did not comply with treatment and testing protocols and were withdrawn from the analysis (PCSO-524® *n* = 2). Ten interstate participants were lost to follow-up and failed to return any data (PCSO-524® *n* = 5). Compliance rates were determined via a pill count, the compliance diary and a follow-up telephone call. The mean recorded compliance rates with the single daily intake were 96.67%. There were no significant differences between compliance, diagnosis and ADHD medication between the treatment groups.

## Discussion

The results of this study did not support the hypothesis that PCSO-524® would improve parent-rated levels of hyperactivity, inattention and impulsivity in children with high levels of behavioural problems aged 6 to 14 years over placebo. Despite this, several positive findings in exploratory subgroup analyses revealed specific benefits of PCSO-524® in ameliorating the symptoms of hyperactivity and inattention in children who did not meet criteria for parental ratings of combined hyperactivity and inattention. In addition, those children consuming PCSO-524**®** demonstrated significant memory improvements on cognitive tasks against placebo, regardless of symptom severity. The present study utilised parents’ ratings to understand how the symptoms present in each child affected their behaviour at home and at school, regardless of their clinical diagnosis. This type of exploratory subsample analysis allows researchers to understand the benefits of unique treatments such as PCSO-524® and has been utilised previously in rigorous intervention trials in child and adolescent populations (Manor et al. 2012).

Significant improvements in hyperactivity, inattention and learning in the NCT group highlight the possibility of mineral deficiencies in the subsample population under study. This is consistent with previous research indicating parental ratings of children’s behaviour correlates with mineral deficiencies (Sinn et al. 2008) as well as prevalence rates of ADHD (Visser et al. 2013). This also highlights the possibility for future dose range investigations of this extract (PCSO-524®) in order to determine whether children classified as combined type require a larger dose of the intervention or if unrelated factors lead to the lack of improvement in behavioural ratings. The Conners Rating Scales are among the most validated test instruments used in research on ADHD and subclinical levels of ADHD (Archer and Newsom 2000). Hurtig et al. (2007) investigated the presence and severity of ADHD symptoms in a cohort of Finnish children and adolescents in whom ADHD had been diagnosed. Findings indicated that those children who had a combined subtype diagnosis had significantly worse problems of inattention, compared with children who had the inattentive subtype alone. This may indicate as to why the NCT children in the current trial benefited more from the treatment intervention than those who had a CT outcome. Furthermore, children within the NCT subsample who displayed levels of high inattention only demonstrated greater benefit due to the intervention than those children classified as high hyperactivity only or non-subtype. This outcome supports a previous study by Johnson et al. (2009) that demonstrated that responders to a combination of lipids were more likely to be male and have a diagnosis of ADHD inattentive subtype. Gillies et al. (2012) concluded that combined omega-3 and omega-6 PUFA supplements may be more likely to improve ADHD-associated symptoms than omega-3 supplementation alone. Previous biochemical reports have highlighted that the potent anti-inflammatory properties of *P. canaliculus* may be due to the synergistic action of multiple PUFAs, rather than the omega-3 (DHA) content by itself (Treschow et al. 2007).

The significant improvements in whole sample COMPASS scores on measures of picture recognition indicate that PCSO-524® may improve elements of delayed working memory. In a study that investigated omega-3 supplementation in young healthy adults, improvements were seen in terms of memory and reaction times, which complemented the improved cognitive results reported here (Stonehouse et al. 2013). Cognitive changes across different intervention studies are often difficult to compare with different measures often used. A recent approach to better understand the cognitive domains measured by different tests has been suggested by Pase and Stough (2014), who have advocated for the use of the Cattell-Horn-Carroll (CHC) cognitive framework for interventions. Future studies should use the CHC as a method to understand which cognitive domains have been tested from study to study.

In clinical and subclinical domains, externalised symptoms such as hyperactivity and inattention require accurate measures by which research can detect changes reliably. Continuous performance tests (CPTs) offer crucial insight into issues with inattention and impulsivity. Greenberg and Waldman (1993) established the normative data for the TOVA (formerly the Minnesota Computer Assessment; Greenberg 1987) in a 1993 publication that used data collected from 775 children aged 6 to 16. In the present study, the results from the TOVA testing assessed the accuracy and speed at which participants recovered after they made an error, which was denoted as *post commission.* In a consistent trend, children without ADHD who consumed PCSO-524® demonstrated an improved ability to respond faster to targets consistently following an incorrect response. A previous study by Vaisman et al. (2008) suggested that interventions with greater EPA/DHA ratios, even in subgram amounts, could impact the visual sustained attention performance in paediatric populations. Despite promising subsample outcomes, whole sample analysis of the TOVA results did not demonstrate significant improvements in either treatment group.

Neurochemical explanations for improvements in hyperactivity, impulsivity and inattention remain difficult to discern. In a study by Ma et al. (2011), researchers established that children who were classified to have ADHD hyperactive/impulsive type had significantly lower cortisol levels than the ADHD combined type and ADHD inattentive-type children. As PCSO-524® is an effective mediator for the 5-LOX and 12-LOX pathways, its presence in this study may have improved levels of cortisol via its effect on cytokines. Cytokines play a key role in the hypothalamic-pituitary-adrenal (HPA) axis and have been shown to be involved in cognitive processes, stress and depression (Wilson et al. 2002). Lower levels of omega-3 (DHA) within the adult brain have been shown to dysregulate the functions of the HPA axis (Vaisman et al. 2008). Lipid profile studies have demonstrated a higher AA/EPA ratio in those children with ADHD (Stonehouse et al. 2013). Improvements in this AA/EPA status has correlated with a decrease in ADHD-associated symptoms (Stonehouse et al. 2013). Germano et al. (2007) evaluated the effects of LC PUFAs in ADHD children against normal controls highlighting a significant correlation between improved clinical symptoms of hyperactivity and inattention with reduced disparity in the AA/EPA ratio. Despite the lower level of omega-3s in PCSO-524®, previous evidence of its ability to block pro-inflammatory pathways highlights a possible mechanism of action via returning the AA/EPA ratio or inflammatory/anti-inflammatory ratio to a more balanced state (Halpern 2000; Lello et al. 2012; Gillies et al. 2012). LC PUFAs improve blood lipid profiles, cardiovascular health, cell membrane fluidity and cell signalling cascades, so the introduction of any amount of LC PUFAs into a cell system that has low levels of it may improve behaviours which are affected by its absence. The most prevalent fatty acid within the brain is DHA, which constitutes 45 to 65% of fatty acids in nervous tissues (Ma et al. 2011). There were no significant differences in compliance, omega-3 intake through food or reporting of symptoms between treatment groups, and these results may indicate an increased bioavailability of omega-3s in the brain following PCSO-524® supplementation.

Limitations of the current study include the broad spectrum of participants. Despite the large sample size, the variety in participant demographics, diagnostic status and locations may hinder the extrapolation of the results. Although it does not directly impact the outcome of the data collected, the small number of participants’ families who failed to provide medication information diminishes any inferences that could be made regarding medication-specific adjunct treatment. The significant number of the males in the study may highlight a well-known bias in the wider population; males with ADHD tend to exhibit more externalising behaviours such as hyperactivity, inattention and impulsivity, whereas females with ADHD tend to exhibit more social issues and internalising comorbidities (anxiety) (Gershon and Gershon 2002; Carlson et al. 1997). This bias may impact the reasons parents decide to join the study as well as explain the greater number of males over females recruited. The outcome of the male-only analysis indicates that PCSO-524® may be beneficial for young males with issues of hyperactivity or inattention at the subclinical level. Further investigations need to be done to verify this outcome.

## Conclusions

The primary outcome of the current study was not supported by the results. Replicated randomised trials with dose variations and lipid profiling are needed to understand the neurological and behavioural benefits of PCSO-524® in this population. Despite this, exploratory post hoc analysis of the primary outcome of the current study indicates promise for the use of PCSO-524® in the treatment of symptoms of inattention, hyperactivity and impulsivity in children and adolescents with or without ADHD displaying less severe behavioural symptoms. PCSO-524® also indicated an ability to improve working memory in children and adolescents with increased levels of hyperactivity, inattention and impulsivity. Post hoc analysis of those children with a diagnosis of ADHD also demonstrated improved inhibition and reduced error making compared to placebo. The use of PCSO-524® as an adjunctive treatment should be subjected to additional clinical trials (i.e. together with stimulant medication). Further large-scale RCTs should be conducted administering PCSO-524® to children and adolescents with subclinical levels of hyperactivity inattention and impulsivity. Future neuroimaging trials may assist in elucidating the mechanism of action.

## Clinical significance

PCSO-524®, a marine-based LC PUFA, may be a safe alternative to standard pharmaceutical treatments for children and adolescents with ADHD who have less severe levels of hyperactivity, inattention and impulsivity.

## References

[CR1] American Psychiatric Association (2000). Diagnostic and statistical manual of mental disorders DSM-IV-TR.

[CR2] Archer RP, Newsom CR (2000). Psychological test usage with adolescent clients: survey update. Assessment.

[CR3] Balázs J, Keresztény Á (2014) Subthreshold attention deficit hyperactivity in children and adolescents: a systematic review. Eur Child Adolesc Psychiatry 1–16. doi:10.1007/s00787-013-0514-7 10.1007/s00787-013-0514-7 24399038

[CR4] Biederman J (2005). Attention-deficit/hyperactivity disorder: a selective overview. Biol Psychiatry.

[CR5] Bloch MH, Qawasmi A (2011). Omega-3 fatty acid supplementation for the treatment of children with attention deficit/hyperactivity disorder symptomatology: systematic review and meta-analysis. J Am Acad Child Adolesc Psychiatry.

[CR6] Bos DJ, Oranje B, Veerhoek ES, Van Diepen RM, Wusten JMH, Demmelmair H, Koletzko B, Sain-van der Velden MGM, Eilander A, Hoeksma M, Durston S (2015). Reduced symptoms of inattention after dietary omega 3 fatty acid supplementation in boys with and without attention deficit/hyperactivity disorder. Neuropsychopharmacology.

[CR7] Busner J, Targum SD (2007). The clinical global impressions scale: applying a research tool in clinical practice. Psychiatry (Edgmont).

[CR8] Carlson CL, Tamm L, Gaub M (1997). Gender differences in children with ADHD, ODD, and Co-occurring ADHD/ODD identified in a school population. Journal of the American Academy of Child & Adolescent Psychiatry.

[CR9] Clarke AR, Barry RJ, McCarthy R, Selikowitz M (2001). Electroencephalogram differences in two subtypes of attention-deficit/hyperactivity disorder. Psychophysiology.

[CR10] Conners CK, Sitarenios G, Parker JDA, Epstein JN (1998). The revised Conners’ parent rating scale (CPRS-R): factor structure, reliability, and criterion validity. J Abnorm Child Psychol.

[CR11] Dugas B (2000). Lyprinol inhibits LTB4 production by human monocytes. Allerg Immunol.

[CR12] DuPaul GJ, Power TJ, Anastopoulos AD, R R (1998). ADHD rating scale-IV: checklists, norms, and clinical interpretation.

[CR13] Emelyanov A, Fedoseev G, Krasnoschekova O (2002). Treatment of asthma with lipid extract of New Zealand green-lipped mussel: a randomised clinical trial. Eur Respir J.

[CR14] Faries DE, Yalcin I, Harder D, Heiligenstein JH (2001). Validation of the ADHD rating scale as a clirlician administered and scored instrument. J Atten Disord.

[CR15] Gadit AA (2003). Subthreshold mental disorders. *JPMA*. The Journal of the Pakistan Medical Association.

[CR16] Germano M, Meleleo D, Montorfano G (2007). Plasma, red blood cells phospholipids and clinical evaluation after long chain omega-3 supplementation in children with attention deficit hyperactivity disorder (ADHD). Nutr Neurosci.

[CR17] Gershon J, Gershon J (2002). A meta-analytic review of gender differences in ADHD. J Atten Disord.

[CR18] Gillies D, Sinn J, Lad SS, Leach MJ, Ross MJ (2012). Polyunsaturated fatty acids (PUFA) for attention deficit hyperactivity disorder (ADHD) in children and adolescents. Cochrane Database Syst Rev.

[CR19] Grayson DS, Kroenke CD, Neuringer M, Fair DA (2014). Dietary omega-3 fatty acids modulate large-scale systems organization in the rhesus macaque brain. The Journal of neuroscience : the official journal of the Society for Neuroscience.

[CR20] Greenberg LM (1987). An objective measure of methylphenidate response: clinical use of the MCA. Psychopharmacol Bull.

[CR21] Greenberg LM, Waldman ID (1993). Developmental normative data on the test of variables of attention (T.O.V.A.). Journal of child psychology and psychiatry, and allied disciplines..

[CR22] Halpern GM (2000). Anti-inflammatory effects of a stabilized lipid extract of Perna canaliculus (lyprinol). Allerg Immunol.

[CR23] Hariri M, Djazayery A, Djalali M, Saedisomeolia A, Rahimi A, Abdolahian E (2012). Effect of n-3 supplementation on hyperactivity, oxidative stress and inflammatory mediators in children with attention-deficit-hyperactivity disorder. Malays J Nutr.

[CR24] Hurtig T, Ebeling H, Taanila A (2007). ADHD symptoms and subtypes: relationship between childhood and adolescent symptoms. J Am Acad Child Adolesc Psychiatry.

[CR25] Johnson M, Ostlund S, Fransson G, Kadesjo B, Gillberg C (2009). Omega-3/omega-6 fatty acids for attention deficit hyperactivity disorder: a randomized placebo-controlled trial in children and adolescents. J Atten Disord.

[CR26] Kalafatis N (1996) Theodore, Inventor; Pharmalink International Limited, assignee. Lipid extract having anti-inflammatory activity

[CR27] Kean JD, Camfield D, Sarris J (2013). A randomized controlled trial investigating the effects of PCSO-524(R), a patented oil extract of the New Zealand green lipped mussel (perna canaliculus), on the behaviour, mood, cognition and neurophysiology of children and adolescents (aged 6--14 years) experiencing clinical and sub-clinical levels of hyperactivity and inattention: study protocol ACTRN12610000978066. Nutr J.

[CR28] Kobor A, Takacs A, Urban R, Csepe V (2012). The latent classes of subclinical ADHD symptoms: convergences of multiple informant reports. Res Dev Disabil.

[CR29] Kohlboeck G, Glaser C, Tiesler C (2011). Effect of fatty acid status in cord blood serum on children’s behavioral difficulties at 10 y of age: results from the LISAplus study. Am J Clin Nutr.

[CR30] Lello J, Liang A, Robinson E, Leutenegger D, Wheat A (2012) Treatment Of children’s asthma with a lipid extract of the New Zealand green lipped mussel (*Perna canaliculus*) (Lyprinol®) - a double blind, randomised controlled trial in children with moderate to severe chronic obstructive asthma. The Internet Journal of Asthma, Allergy and Immunology 8(1)

[CR31] Llorente AM, Voigt R, Jensen CL, Fraley JK, Heird WC, Rennie KM (2008). The test of variables of attention (TOVA): internal consistency (Q 1 vs. Q2 and Q3 vs. Q4) in children with attention deficit/hyperactivity disorder (ADHD). Child Neuropsychology.

[CR32] Ma L, Chen YH, Chen H, Liu YY, Wang YX (2011). The function of hypothalamus-pituitary-adrenal axis in children with ADHD. Brain Res.

[CR33] Malmberg K, Edbom T, Wargelius HL, Larsson JO (2011). Psychiatric problems associated with subthreshold ADHD and disruptive behaviour diagnoses in teenagers. Acta paediatrica (Oslo, Norway : 1992).

[CR34] Mann CA, Lubar JF, Zimmerman AW, Miller CA, Muenchen RA (1992). Quantitative analysis of EEG in boys with attention-deficit-hyperactivity disorder: controlled study with clinical implications. Pediatr Neurol.

[CR35] Manor I, Magen A, Keidar D (2012). The effect of phosphatidylserine containing Omega3 fatty-acids on attention-deficit hyperactivity disorder symptoms in children: a double-blind placebo-controlled trial, followed by an open-label extension. Eur Psychiatry.

[CR36] Mickleborough TD, Vaughn CL, Shei RJ, Davis EM, Wilhite DP (2013). Marine lipid fraction PCSO-524 (lyprinol/omega XL) of the New Zealand green lipped mussel attenuates hyperpnea-induced bronchoconstriction in asthma. Respir Med.

[CR37] Pase M, Stough C (2014). An evidence-based method for examining and reporting cognitive processes in nutrition research. Nutr Res Rev.

[CR38] Richardson AJ (2006). Omega-3 fatty acids in ADHD and related neurodevelopmental disorders. International Review of Psychiatry.

[CR39] Rielly NE, Craig WM, Parker KC (2006). Peer and parenting characteristics of boys and girls with subclinical attention problems. J Atten Disord.

[CR40] Scholey A, Ossoukhova A, Owen L (2010). Effects of American ginseng (*Panax quinquefolius*) on neurocognitive function: an acute, randomised, double-blind, placebo-controlled, crossover study. Psychopharmacology.

[CR41] Shankman SA, Lewinsohn PM, Klein DN, Small JW, Seeley JR, Altman SE (2009). Subthreshold conditions as precursors for full syndrome disorders: a 15-year longitudinal study of multiple diagnostic classes. Journal of child psychology and psychiatry, and allied disciplines.

[CR42] Sinn N, Bryan J (2007). Effect of supplementation with polyunsaturated fatty acids and micronutrients on learning and behavior problems associated with child ADHD. J Dev Behav Pediatr.

[CR43] Sinn N, Bryan J, Wilson C (2008). Cognitive effects of polyunsaturated fatty acids in children with attention deficit hyperactivity disorder symptoms: a randomised controlled trial. Prostaglandins Leukotrienes and Essential Fatty Acids.

[CR44] Sonuga-Barke EJS, Brandeis D, Cortese S, Daley D, Ferrin M, Holtmann M (2013). Nonpharmacological intervenions for ADHD: systematic review and meta-analyses of randomized controlled trials of dietary and psychological treatments. Am J Psychiatr.

[CR45] Sorgi PJ, Hallowell EM, Hutchins HL, Sears B (2007) Effects of an open-label pilot study with high-dose EPA/DHA concentrates on plasma phospholipids and behavior in children with attention deficit hyperactivity disorder. Nutr J 6 10.1186/1475-2891-6-16 PMC1971271 17629918

[CR46] Stonehouse W, Conlon CA, Podd J (2013). DHA supplementation improved both memory and reaction time in healthy young adults: a randomized controlled trial. Am J Clin Nutr.

[CR47] Tenikoff D, Murphy KJ, Le M, Howe PR, Howarth GS (2005). Lyprinol (stabilised lipid extract of New Zealand green-lipped mussel): a potential preventative treatment modality for inflammatory bowel disease. J Gastroenterol.

[CR48] Terry PC, Lane AM, Lane HJ, Keohane L (1999). Development and validation of a mood measure for adolescents. J Sports Sci.

[CR49] Vaisman N, Kaysar N, Zaruk-Adasha Y (2008). Correlation between changes in blood fatty acid composition and visual sustained attention performance in children with inattention: effect of dietary n-3 fatty acids containing phospholipids. Am J Clin Nutr.

[CR50] Visser SN, Danielson ML, Bitsko RH, Perou R, Blumberg SJ (2013). Convergent validity of parent-reported attention-deficit/hyperactivity disorder diagnosis: a cross-study comparison. JAMA Pediatr.

[CR51] Wehmeier PM, Schacht A, Barkley RA (2010). Social and emotional impairment in children and adolescents with ADHD and the impact on quality of life. J Adolesc Health.

[CR52] Whitehouse MW, Rainsford KD (2006). Lipoxygenase inhibition: the neglected frontier for regulating chronic inflammation and pain. Infammapharmacology.

[CR53] Whitehouse MW, Macrides TA, Kalafatis N (1997). Anti-inflammatory activity of a lipid fraction (lyprinol) from the NZ green-lipped mussel. Infammopharmacology.

[CR54] Wilson CJ, Finch CE, Cohen HJ (2002). Cytokines and cognition—the case for a head-to-toe inflammatory paradigm. J Am Geriatr Soc.

[CR56] Wolraich ML, Wibbelsman CJ, Brown TE (2005). Attention-deficit/hyperactivity disorder among adolescents: a review of the diagnosis, treatment, and clinical implications. Pediatrics.

[CR57] World Medical Association. (2008) Declaration of Helsinki. Ethical principles for medical research involving human subjects. http://www.wma.net/e/policy/b3.htm. 19886379

[CR58] Young GS, Conquer JA, Thomas R (2005). Effect of randomized supplementation with high dose olive, flax or fish oil on serum phospholipid fatty acid levels in adults with attention deficit hyperactivity disorder. Reprod Nutr Dev.

[CR59] Zawadzki M, Janosch C, Szechinski J (2013). Perna canaliculus lipid complex PCSO-524™ demonstrated pain relief for osteoarthritis patients benchmarked against fish oil, a randomized trial, without placebo control. Marine Drugs.

